# Monitoring carbohydrate 3D structure quality with the *Privateer* database

**DOI:** 10.3762/bjoc.20.83

**Published:** 2024-04-24

**Authors:** Jordan S Dialpuri, Haroldas Bagdonas, Lucy C Schofield, Phuong Thao Pham, Lou Holland, Jon Agirre

**Affiliations:** 1 York Structural Biology Laboratory, Department of Chemistry, University of York, UK

**Keywords:** carbohydrates, database, *N*-glycans, *N*-glycosylation, polysaccharides, validation, website

## Abstract

The remediation of the carbohydrate data of the Protein Data Bank (PDB) has brought numerous enhancements to the findability and interpretability of deposited glycan structures, yet crucial quality indicators are either missing or hard to find on the PDB pages. Without a way to access wider glycochemical context, problematic structures may be taken as fact by keen but inexperienced scientists. The *Privateer* software is a validation and analysis tool that provides access to a number of metrics and links to external experimental resources, allowing users to evaluate structures using carbohydrate-specific methods. Here, we present the *Privateer* database, a free resource that aims to complement the growing glycan content of the PDB.

## Introduction

Carbohydrate modelling is an important but often cumbersome stage in the macromolecular X-ray structure solution workflow. The accurate modelling of glycoproteins and protein–carbohydrate complexes is pivotal in understanding the complex biochemical interactions that affect the physiological function of cells [1]. Any mechanistic analysis done with finely grained approaches such as QM/MM [2] relies heavily on the correctness of the starting coordinates. Despite this, carbohydrate models often contain modelling inconsistencies that cannot easily be attributed to known biochemical principles [3]. These inconsistencies cannot solely be attributed to model-building inexperience, as carbohydrate model building is an inherently difficult task, which in the past has been plagued with software related problems from incorrect libraries to incomplete support [4]. Carbohydrates are mobile, highly branched additions to the comparatively rigid protein framework; in macromolecular crystallography, this causes heterogeneity throughout the crystal lattice and, therefore, poorly resolved density regions, whereas in electron cryo-microscopy different conformations and compositions are averaged out during image classification and volume reconstruction [5].

Owing to these difficulties, it is not uncommon to find problematic carbohydrate structures in the Protein Data Bank (PDB), from the initial works of Lütteke, Frank and von der Lieth [6–7], who identified numerous issues affecting nomenclature and linkages (estimated to affect 30% of the structures at the time), to the reports of surprising – or indeed glyco-chemically impossible – linkages in a glycoprotein as pointed out by Crispin and collaborators [8], and more recently the realisation that high-energy ring conformations, a rare event in six-membered pyranosides, were present in ca. 15% of the *N-*glycan components of glycoproteins in the PDB [3]. Many of these findings originated the development of new resources, including services and databases [9–13], and standalone software [14–18]. Among these, the *Privateer* software package has been a key tool for glycoprotein and protein–carbohydrate complex validation: *Privateer* analyses the conformational plausibility of each sugar model [3], checks that structures match the nomenclature used for deposition in the PDB [14], compares glycan compositions to known structures as reported by glycomics (e.g., GlyConnect [19]) and glyco-informatics (e.g., GlyTouCan [20]) databases and repositories [15], and checks how close the overall conformation of *N-*glycans comes to that of validated deposited structures [16].

The PDB-REDO [21] database is a separate resource, albeit linked to the PDB in that the entries that compound PDB-REDO are those original PDB crystallographic entries that included experimental data (i.e., reflection intensities or amplitudes); each entry includes a re-refined, sometimes even re-built to some extent, copy of the original model. These newer versions are produced with state-of-the-art methods, many of which were probably not available at the time of deposition; hence, the quality of the models is expected to improve. Because the methodology included in PDB-REDO had been affected by the lack of automatic support that plagued general purpose crystallographic model building and refinement software [4], carbohydrate-specific methods have been gradually introduced over the years [22–23].

Whilst *Privateer* has been a staple tool in carbohydrate validation, the results of *Privateer* have not been collated in such a way that allows for easy judgement of carbohydrate model quality in the PDB [24]. Providing users with metrics that allow them to make chemically sound conclusions about the model is an important facility, especially for novice users. To allow this to happen readily on PDB distribution sites, we present the *Privateer* database, a freely available, up-to-date collection of validation information for both the PDB and PDB-REDO [21] archives.

## Results and Discussion

### Format of the validation report

**Figure 1:**
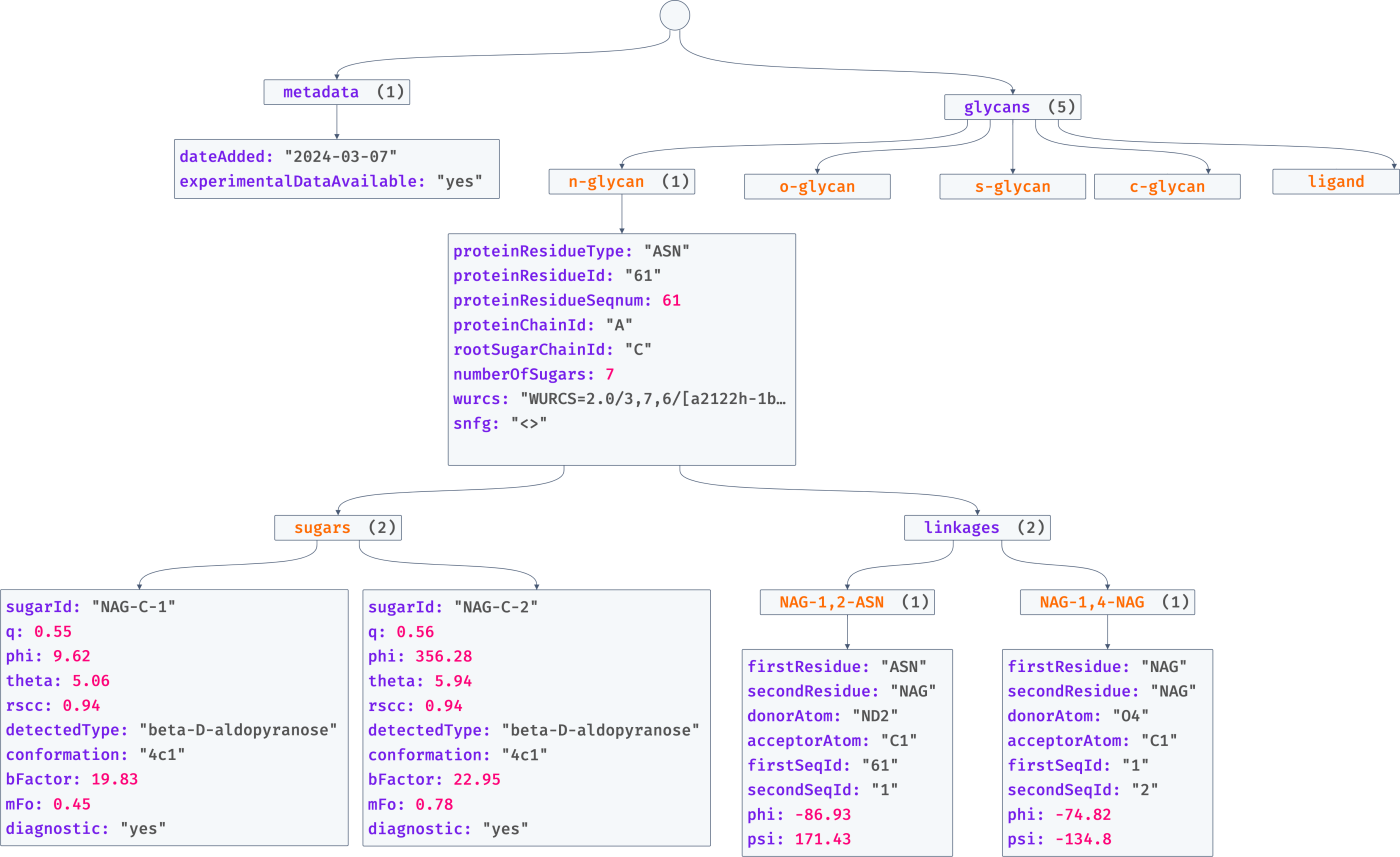
Format of a validation report in JSON format. At the top level of the tree, the report contains metadata about itself, such as the date the entry was added to the database and if experimental data is available. Also at the top level of the tree is the glycan information, separated into glycan types. Each glycan also contains a list of sugars, with a range of validation information and a list of linkage with torsion angle information. Tree visualisation was created with jsoncrack.com.

The JSON file deposited for each PDB entry follows a consistent format, as shown in Figure 1. At the top level, the file contains metadata about the validation report. This metadata provides the date that the validation report was generated as well as the availability of experimental data. It is helpful to have this information easily accessible as *Privateer* cannot calculate the real space correlation coefficient without experimental data; therefore, programmatic access to further validation metrics could be streamlined, knowing the information is not present.

Also at the top level of the validation report is the beginning of the carbohydrate information, listed as ‘glycans’ in the JSON format. Within this ‘glycan’ scope, information is segmented into glycan types, that is, ‘n-glycan’, ‘o-glycan’, ‘s-glycan’, ‘c-glycan’, and 'ligand'. Each of these glycan types contains an array of individual glycans of that type, and the format of the data inside each of these glycan types is identical.

The data contained in each glycan entry is shown in Table 1. Each entry contains information about the protein chain attachment, the number of sugars in the glycan, the WURCS2.0 code [25], the standard nomenclature for glycan SVG, and an array of sugar entries. The validation data calculated by *Privateer* for each sugar entry is shown in Table 2, and that for each linkage is shown in Table 3.

**Table 1:** Data contained within each glycan entry.

Key	Example	Type

proteinResidueType	ASN	string
proteinResidueId	61	string
proteinResidueSeqnum	61	number
proteinChainId	A	string
rootSugarChainId	C	string
numberOfSugars	7	number
wurcs	WURCS=2.0/3,7,6/…	string
snfg	<svg> … </svg>	string
sugars	see Table 2	array

**Table 2:** Data contained within each sugar entry.

Key	Example	Type

sugarID	NAG-D-1	string
q	0.54	number
phi	303.44	number
theta	6.45	number
rscc	0.922	number
detectedType	beta-ᴅ-aldopyranose	string
conformation	4c1	string
bFactor	22.367	number
mFo	0.421	number
diagnostic	yes	string

**Table 3:** Data contained within each linkage entry.

Key	Example	Type

firstResidue	NAG	string
secondResidue	NAG	string
donorAtom	O4	string
acceptorAtom	C1	string
firstSeqId	1	string
secondSeqId	2	string
phi	−54.91	number
psi	−108.47	number

### Visualising a validation report

While the database is available on GitHub for programmatic access, viewing a validation report entry in plaintext can be difficult, time-consuming and would certainly be a poor experience for the end user. To improve the utility of this database, we have provided a visualisation of the information contained within the validation report for both PDB and PDB-REDO databases, which is available alongside the *Privateer Web App* [26], https://privateer.york.ac.uk/database.

**Figure 2:**
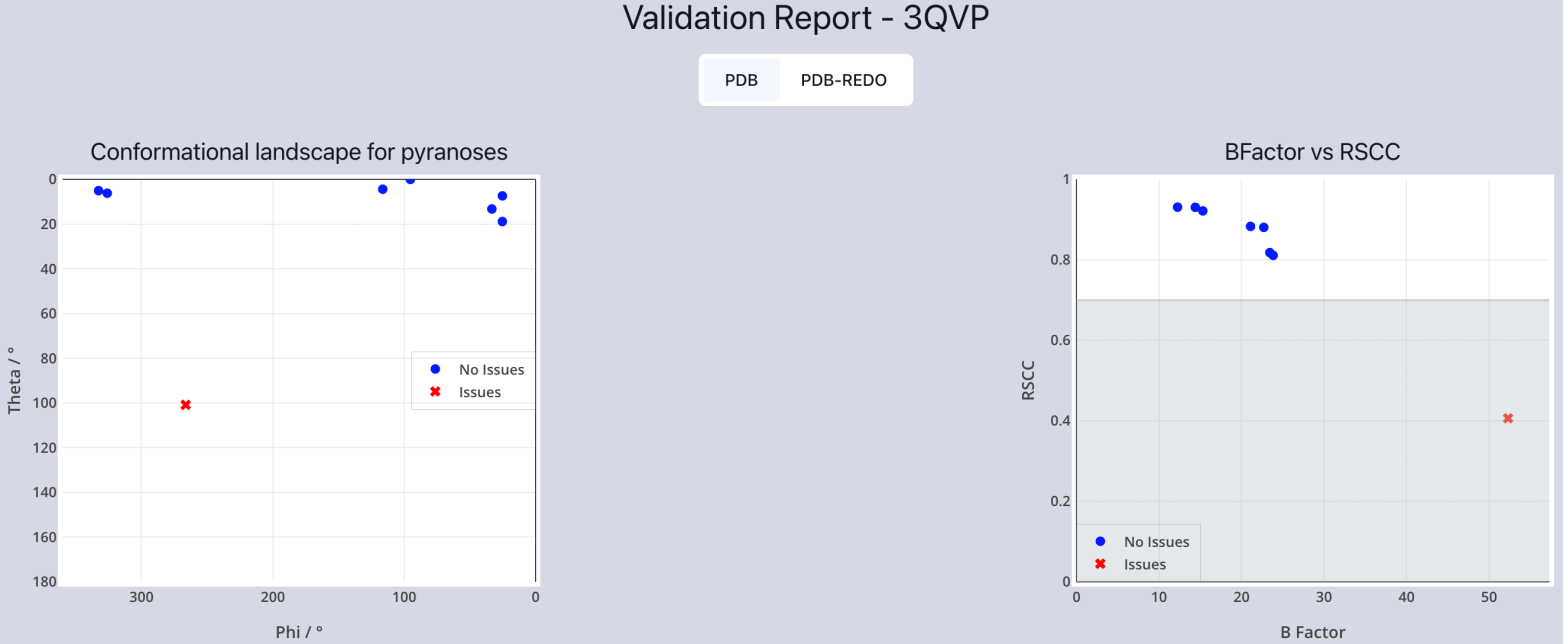
Left: Graphical representation of the conformational landscape of pyranose sugars. A well-modelled ᴅ-sugar would be expected to be in the lowest-energy conformation and have a theta angle close to 0° and would be indicated by a blue point; deviations from the ideal conformation are highlighted with a red cross. Right: Real space correlation coefficient plotted against the B-factor, which enables the refinement of the sugars to be assessed. A slight negative correlation would be expected for a well-refined model. Results taken from the *Privateer* database report for 3QVP [28].

The first section of this visual report displays a global outlook on the validity of the model through two graphs. The first graph shows the conformational landscape for the pyranose sugars. For a sugar model to be deemed valid, the ring must be in the ^4^C_1_ chair conformation. This can be measured through the Cremer–Pople parameters θ and ψ [27]. Theta angles of 0° < θ < 360° indicate that the sugar may be in a higher-energy confirmation; therefore, caution should be placed on any conclusions drawn from the molecular model of the sugar. Also in the first section of the visual validation report is a plot of the B-factor (temperature factor) versus the real space correlation coefficient (RSCC) (Figure 2). A well-refined, well-built model would be expected to have a B-factor that increases somewhat linearly as the RSCC decreases. Over-refined models may deviate from this trend and would be trivial to identify.

**Figure 3:**
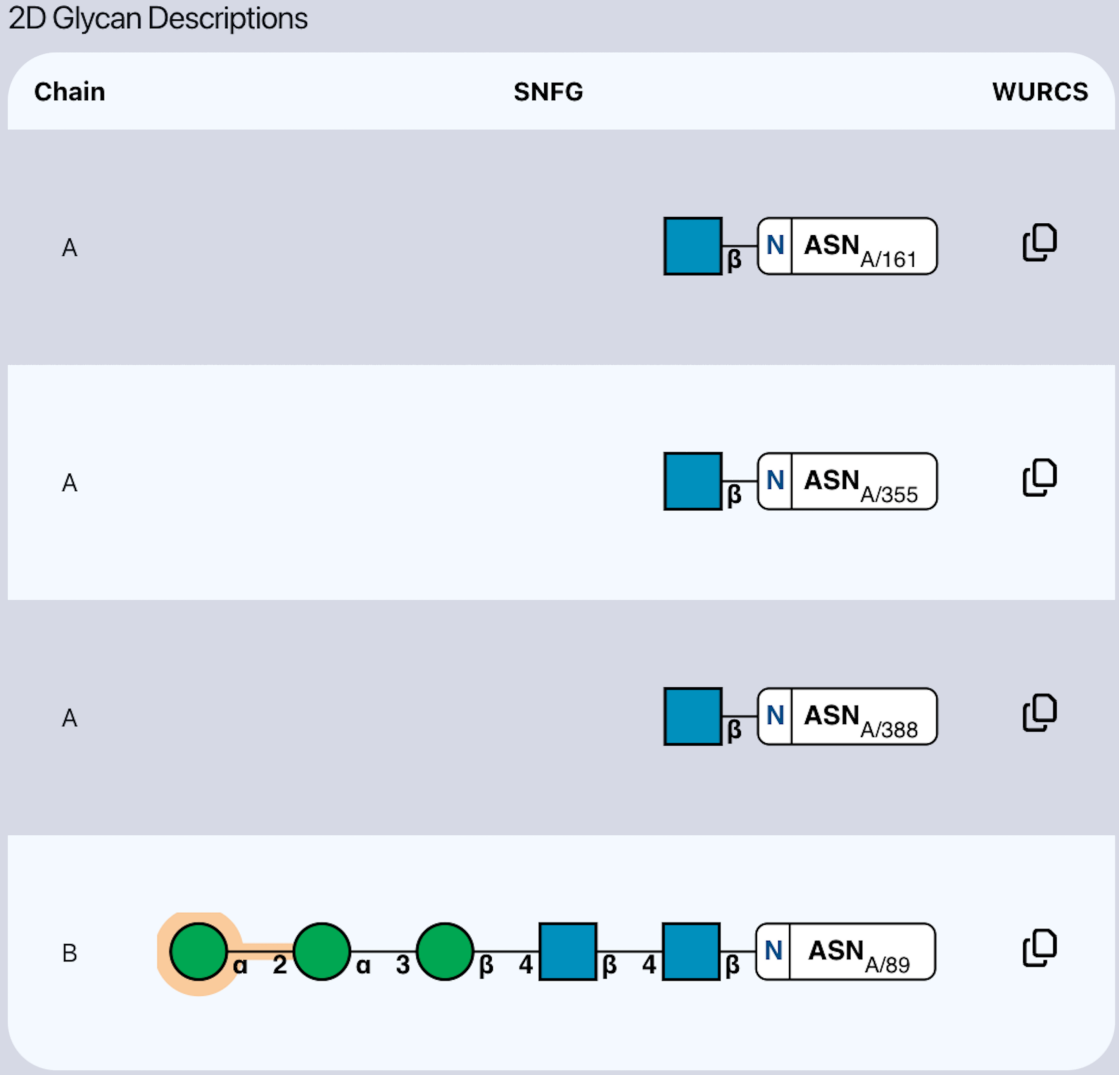
Table of two-dimensional Symbol Nomenclature for Glycan (SNFG) visualisations, which can allow for easy oversight of the validity of a particular glycan. Sugars that have issues identified by *Privateer* are highlighted in orange, and linkages that have unusual torsion angles are also highlighted in orange. The WURCS codes for each glycan are also available to copy to the clipboard. Table taken from the *Privateer* database report for 3QVP.

The validation report also displays a table (Figure 3) representing two-dimensional descriptions of each glycan in the model. Each row in the table represents a unique glycan and includes the chain identifier, standard Symbol Nomenclature for Glycans (SNFG [29]) visualisation, and copyable WURCS [25] identifier. The SNFG displayed for each glycan paints a picture of how well built the glycan model is, as the metrics and validity conclusions calculated by *Privateer* are embedded within each shape and linkage of the diagram. For example, a shape with an orange highlight indicates something is abnormal about the ring’s conformation, puckering, or monosaccharide nomenclature [30]. Similarly, a linkage with an orange highlight indicates that the torsion angles between the linkages are unexpected and require further inspection [16].

In addition to the SNFG, also displayed for each table entry is a copyable WURCS link, which encodes the complete glycan format in a linear code. The decision to present this information as a copyable link, as opposed to as plaintext is due to the inherent difficulty and unlikeliness for a human to read and understand the WURCS code. It is much more likely that the WURCS code would be copied and searched for in a glycomics database, hence we provide that functionality in a streamlined way.

**Figure 4:**
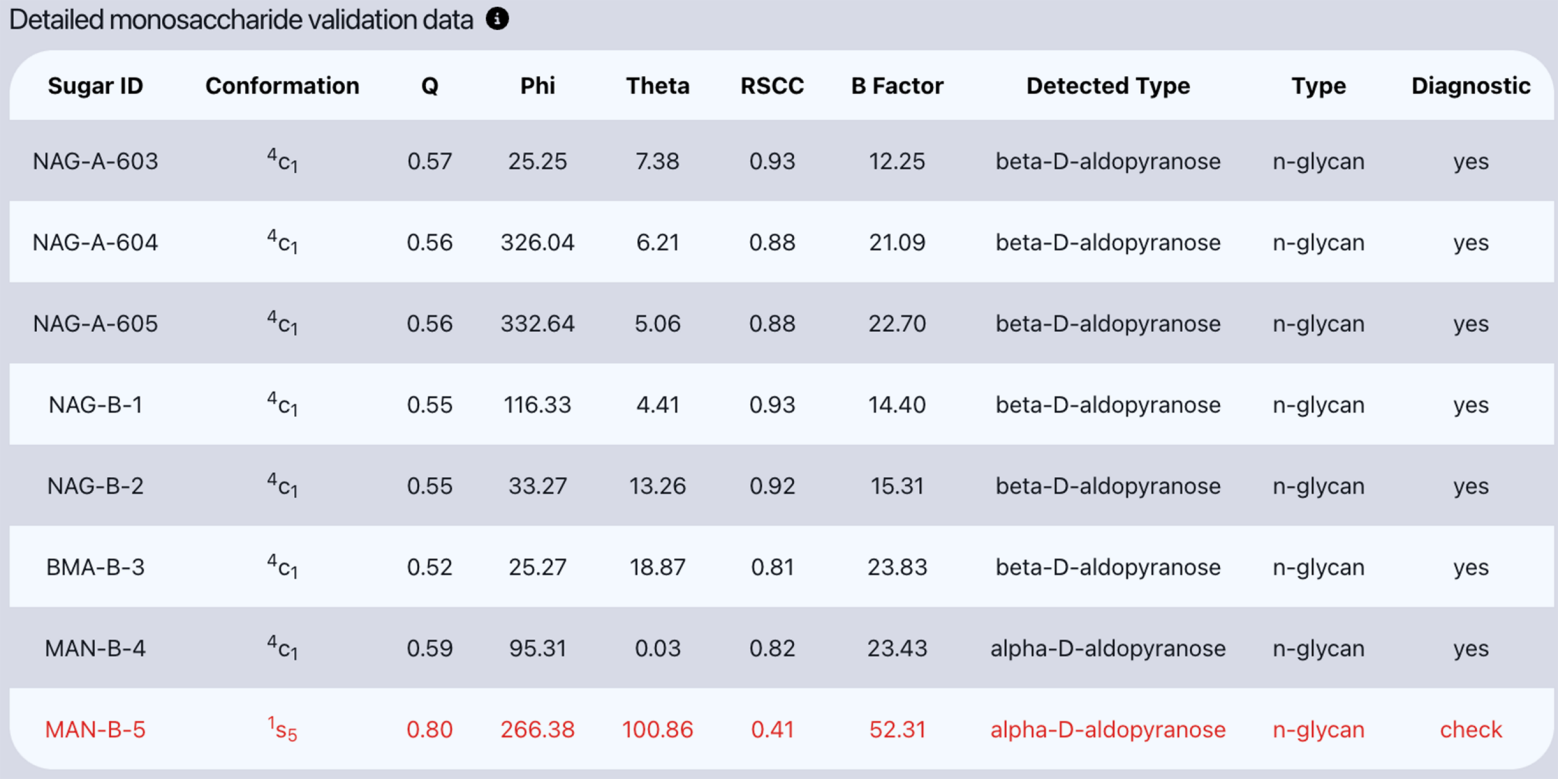
Table of validation data for each sugar residue within PDB code 3QVP available in the visual validation report. The table contains all validation metrics calculated by *Privateer* including the Cremer–Pople puckering parameters, correlation coefficient, and, importantly, *Privateer* diagnostic, which can be used to identify the validity of each sugar. Table taken from the Privateer database report for 3QVP.

The final section of the validation report includes all of the validation metrics calculated by *Privateer* and, most importantly, the diagnostic provided by *Privateer* (Figure 4). A ‘yes’ diagnostic indicates the conformation is correct for the glycosylation type (e.g., ^4^C_1_ for GlcNAc in an *N*-glycan, ^1^C_4_ for mannose in a *C*-glycan), has the correct anomer, and has an acceptable fit to density. This diagnostic indicates that the sugar is valid, whereas a diagnostic of ‘check’ indicates that *Privateer* has detected a potential inconsistency affecting ring conformation, which requires manual inspection. Finally, a ‘no’ diagnostic indicates that the sugar needs a more detailed manual inspection to correct any conformational issues, anomeric issues, or fitting issues.

### Searching for entries in the *Privateer* database

**Figure 5:**
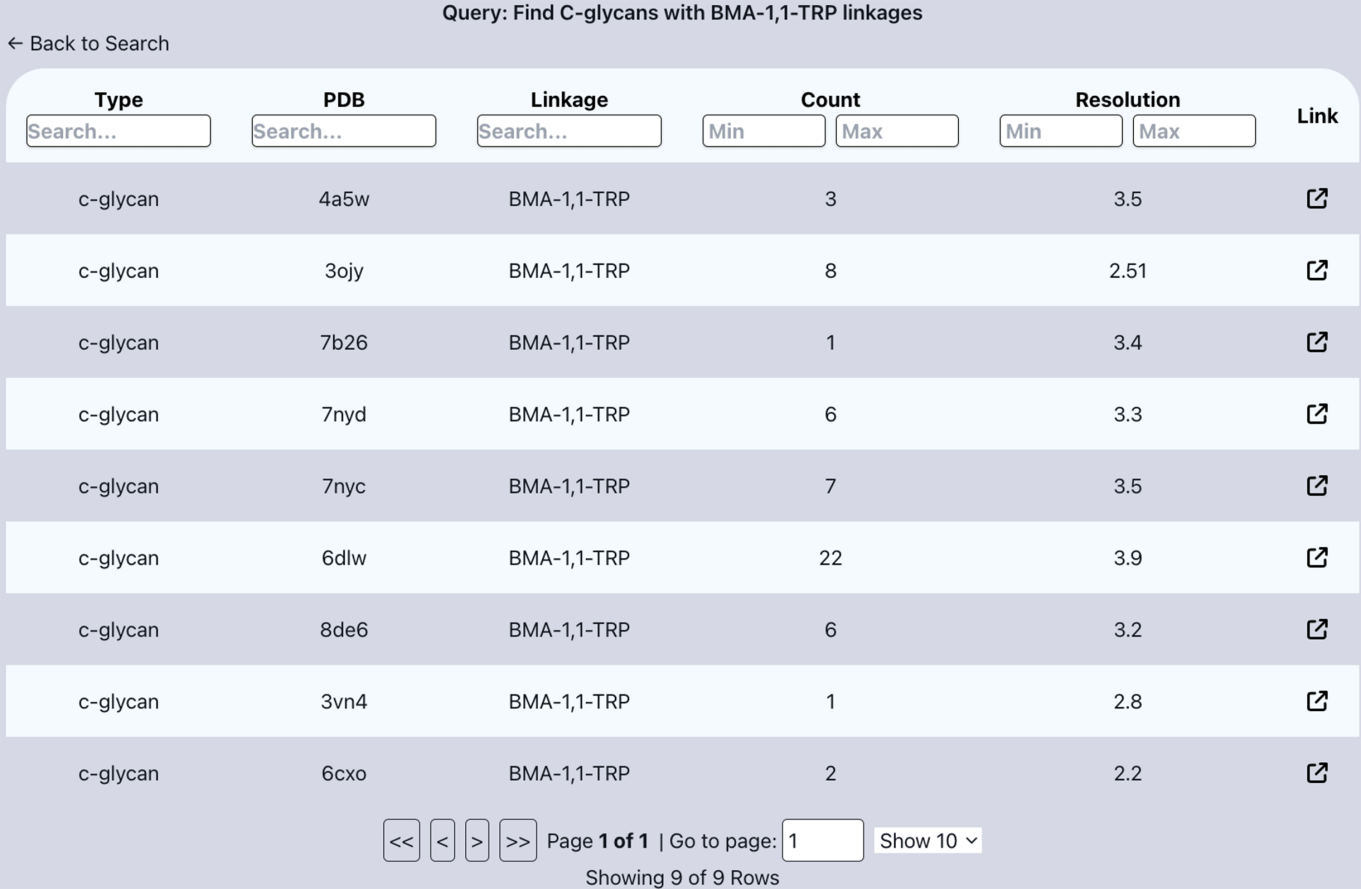
Table of available *Privateer* reports for the BMA-1,1-TRP linkage in *C*-glycans (*C-*mannosylation) sorted by the frequency (count) of the linkage in the deposited model. The table contains information of the carbohydrate type, PDB code, linkage, frequency, and resolution, as well as a link to the *Privateer* database report for each PDB entry.

Another interesting application of the collection of data available in the *Privateer* database is to visualise aggregated carbohydrate data from the PDB. Using the search interface on the *Privateer* database homepage, carbohydrate-containing PDB entries can easily be found and filtered. *Privateer* database entries for specific glycosylation types, namely, *N-*glycosylation, *O*-glycosylation, *S*-glycosylation, or *C*-glycosylation can be filtered quickly and easily. Additional filtering by linkage type is also possible, allowing niche glycosylation targets to be obtained. For example, filtering for *C*-glycans with a ‘BMA-1,1-TRP’ (the correct pair would be ‘MAN-1,1-TRP’, as the linkage in the modification is an alpha linkage) returns nine instances of incorrect sugar conformations in *C*-mannosylation found within the *Privateer* database in a table containing the frequency of the target linkage as well as a link to the *Privateer* database report page for target entry (Figure 5). This table view is also keyword or range-filterable at every data column, which allows for trivial searches of potentially interesting models.

### Trends in the *Privateer* database

**Figure 6:**
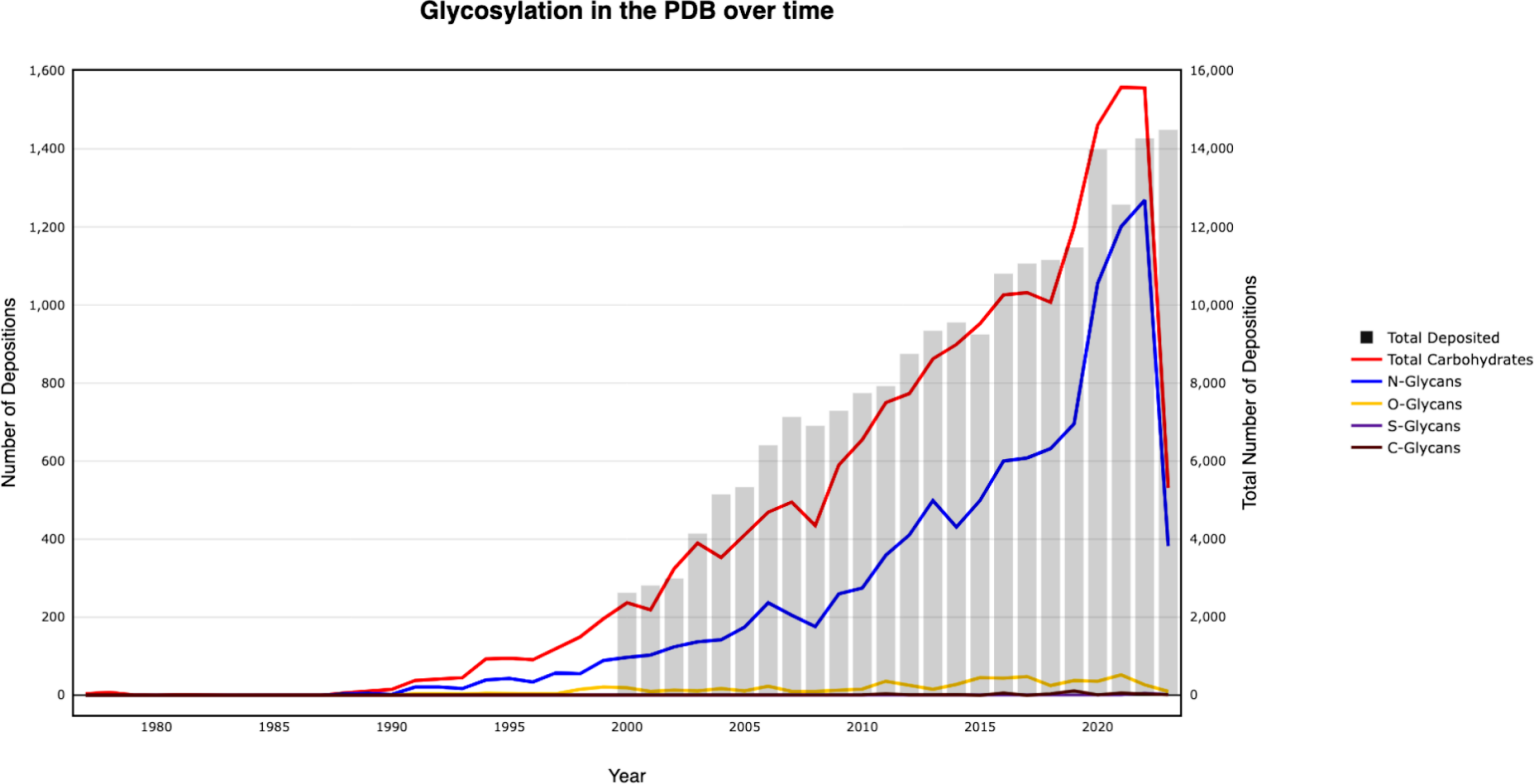
Plot showing trends in deposition in the PDB over time from 1975 to the present. Grey bars show the total deposited models into the PDB for all structural determination methods. Lines show glycosylation in the PDB over time, split into *N*-glycans, *O-*glycans, *S*-glycans, and *C*-glycans.

Using the *Privateer* database, global statistics throughout the PDB and PDB-REDO can be calculated with ease. Observing deposition trends in the PDB is often interesting as it can provide insight into the kinds of structures that are experimentally obtainable over time. With the *Privateer* database, trends in glycosylation deposition in the PDB over time can be measured, as shown in Figure 6. Importantly, as the *Privateer* database is completely recompiled every week, these trends remain consistent with the PDB. To allow for easy and up-to-date observation for anyone, compiled statistics are freely available alongside the *Privateer Web App*, https://privateer.york.ac.uk/statistics.

While simply looking at glycosylation over time using the *Privateer* database is possible, the validation reports calculated by *Privateer* contain a whole host of other interesting pieces of information. In an analogous way to looking at glycosylation over time, the type and validity of carbohydrates in the PDB can also be observed over time. The statistics page available alongside the *Privateer Web App* contains up-to-date plots of validation and conformational errors over time and resolution.

## Conclusion

In conclusion, the new *Privateer* database encompasses the carbohydrate validation capabilities of *Privateer* in an easily accessible pre-prepared form. The database contains all validation metrics calculated by *Privateer* as well as highlighted SNFG diagrams in SVG format for easy third-party web use. Statistics are automatically computed weekly and are available alongside the database both on GitHub and the interactive web page.

## Materials and Methods

The *Privateer* software package [14] was used to compute metrics and statistics for each entry in the PDB [24] or in PDB-REDO [21]. For each structure in the PDB, the carbohydrate-containing chains are first identified before being validated using the suite of validation tools available within *Privateer*. Using the Python bindings available within the latest versions of *Privateer*, a validation report can be generated for each carbohydrate in the molecular model. This report is put out in JSON format for easy consumption by web-based database frontends. The initial report generation was completed in parallel over 64 CPU cores in around 5 h. After the initial surveys through PDB and PDB-REDO, this process only needs to be completed when new molecular models are deposited into the PDB, which occurs weekly. Although compiling validation reports for only new structures would be more efficient, this would fail to encompass changes in structures in historical entries, therefore the *Privateer* database is recompiled weekly.

The database, which receives any updates to the reports after recompilation is hosted on GitHub. The database is separated into PDB and PDB-REDO sections, which are in turn structured in the same format as the PDB archive, separated into folders by the middle two characters of the PDB four-letter code. For convenience, the presentation of the database is hosted alongside the *Privateer Web App* [26]; the database part can be accessed at https://privateer.york.ac.uk/database or by navigating to the database icon on the top right of the screen. The website is dynamic and compatible with desktop and laptop computers, plus tablets and smartphones.

## Data Availability

All source code is publicly available on GitHub (https://github.com/glycojones/privateer and https://github.com/Dialpuri/PrivateerDatabase). The *Privateer* database is available at https://privateer.york.ac.uk/database and calculated statistics are available at https://privateer.york.ac.uk/statistics. Both pages will remain automatically updated with respect to the source code on GitHub.
